# Cerebellar grey matter volume is associated with semantic fluency performance in amyotrophic lateral sclerosis patients

**DOI:** 10.1093/braincomms/fcaf230

**Published:** 2025-06-12

**Authors:** Annaliis Lehto, Julia Schumacher, Stefan Teipel, Judith Machts, Stefan Vielhaber, Andreas Hermann, Johannes Prudlo, Elisabeth Kasper

**Affiliations:** Translational Neurodegeneration Section ‘Albrecht Kossel’, Department of Neurology, Rostock University Medical Center, Rostock 18147, Germany; Deutsches Zentrum für Neurodegenerative Erkrankungen (DZNE), Rostock-Greifswald, Rostock 18147, Germany; Deutsches Zentrum für Neurodegenerative Erkrankungen (DZNE), Rostock-Greifswald, Rostock 18147, Germany; Department of Neurology, Rostock University Medical Center, Rostock 18147, Germany; Deutsches Zentrum für Neurodegenerative Erkrankungen (DZNE), Rostock-Greifswald, Rostock 18147, Germany; Department of Psychosomatic Medicine, Rostock University Medical Center, Rostock 18147, Germany; Otto von Guericke University Magdeburg, Department of Neurology, Magdeburg 39120, Germany; Otto von Guericke University Magdeburg, Department of Neurology, Magdeburg 39120, Germany; Translational Neurodegeneration Section ‘Albrecht Kossel’, Department of Neurology, Rostock University Medical Center, Rostock 18147, Germany; Deutsches Zentrum für Neurodegenerative Erkrankungen (DZNE), Rostock-Greifswald, Rostock 18147, Germany; Deutsches Zentrum für Neurodegenerative Erkrankungen (DZNE), Rostock-Greifswald, Rostock 18147, Germany; Department of Neurology, Rostock University Medical Center, Rostock 18147, Germany; Deutsches Zentrum für Neurodegenerative Erkrankungen (DZNE), Rostock-Greifswald, Rostock 18147, Germany; Department of Neurology, Rostock University Medical Center, Rostock 18147, Germany

**Keywords:** amyotrophic lateral sclerosis, cerebellum, cognition, grey matter volume, diffusion tensor imaging

## Abstract

The cerebellum has been shown to contribute to different cognitive functions such as verbal fluency and different aspects of executive functioning, which are also commonly impaired in amyotrophic lateral sclerosis (ALS) patients. Whereas cerebellar involvement has been indicated in ALS patients in general, its relative contribution to the patients’ specific cognitive deficits remains unclear. In the current analyses, the demographic, clinical, neuropsychological and imaging data of 120 ALS patients and 88 healthy controls were analysed. Grey matter volume (GMV) and white matter (WM) fractional anisotropy were extracted for a comprehensive list of cerebral and cerebellar regions and bootstrapped elastic net regularized regression analyses were employed to identify regional structural metrics that were related to various cognitive scores. We further examined the stability of predictor variables selection and the regression coefficient distributions across the bootstrap samples. Both regional GMV and WM integrity are featured as informative predictors for patients’ cognitive scores. The GMV of cerebellar lobules V and VIIIa were related to semantic fluency, but cerebellar regions did not reliably contribute to other cognitive outcomes. The GMV of pallidum was positively correlated with fluency outcomes and working memory, whereas hippocampus volume was positively related to fluency and episodic memory outcomes. Unsurprisingly, educational achievement emerged as the most general and reliable predictor of cognitive performance. Based on the current findings, cerebellar GMV seems to be specifically associated with semantic fluency performance in ALS patients but not any of the other cognitive measures. Further cognitive functions were associated with both cerebral grey matter (GM) and WM metrics. Future investigations could examine the possible involvement of the cerebellum in the affective and social-emotional dysfunction present in a subset of ALS patients.

## Teaser

Lehto et al. found that the grey matter volume of cerebellar lobules was related to semantic fluency performance in amyotrophic lateral sclerosis patients. The patients' performance on other cognitive tasks was related to cerebral but not cerebellar grey matter and white matter metrics.

## Graphical Abstract

**Graphical Abstract:**
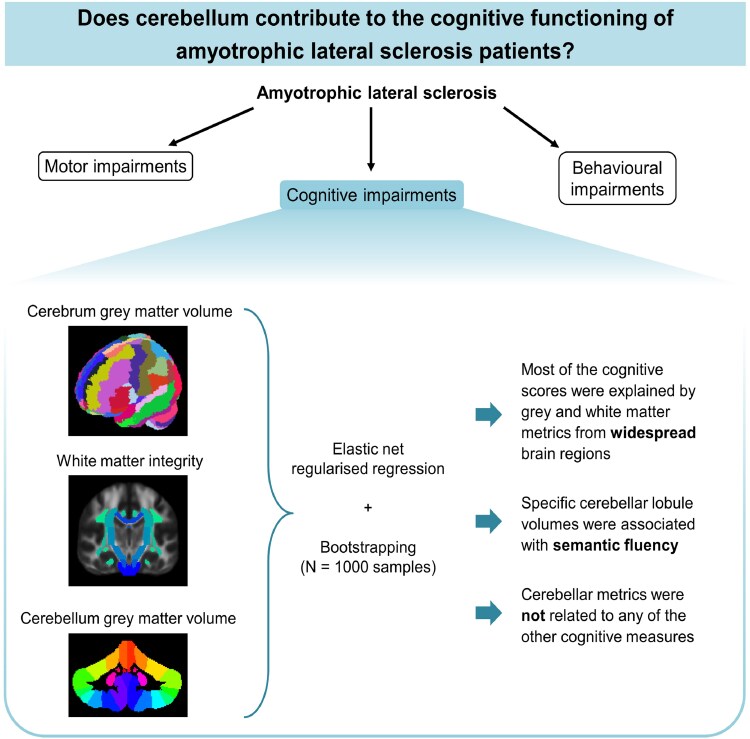

## Introduction

Over the last decades, various studies have investigated the role of the cerebellum in cognition, and established its involvement in a wide range of cognitive and affective processes besides its already well-characterized contribution to motor control. Specialized cerebrocerebellar circuits connect the cerebellum with widespread cortical and subcortical areas^1-3^ and the cerebellum’s main function has been proposed to be encoding sequence and timing information.^4,5^ In accordance, cerebellar contribution has been demonstrated especially for cognitive tasks requiring strategic thinking, planning and verbal or non-verbal associative learning.^5^ Nevertheless, different neurobehavioural, neurophysiological and neuroimaging studies over the last four decades have reported cerebellar involvement in countless other cognitive tasks.^6-8^ Moreover, in recognition of non-motor deficits after cerebellar lesions, the cerebellar cognitive affective syndrome has been coined.^9-11^ Cognition arises from the coordinated activity of a wider network of brain regions and the compiling evidence points to the inclusion of the cerebellum in such a network.

Amyotrophic lateral sclerosis (ALS) is a neurodegenerative disease that affects upper and lower motor neurons and is prominently characterized by progressive muscular atrophy. ALS has increasingly been recognized as a multisystem disorder with diverse clinical manifestations and widespread neuropathological changes. Besides rapidly advancing motor impairment, up to 50% of people living with ALS have mild cognitive deficits^12^ and the characteristically affected domains include verbal fluency, executive functioning, social cognition and language.^13,14^ Various studies have indicated cerebellar involvement in ALS based on grey (GM) and white matter (WM) structural changes^15-21^ and increases in glucose metabolism.^22-24^ The literature on structural cerebellar changes in ALS, however, is highly inconsistent in the location and extent of changes, and few studies have examined cerebellar changes on the level of individual lobules or peduncles.^25^

As cerebellar involvement has been indicated in ALS in general, we aimed to investigate its relative contribution in comparison with cerebral changes to the cognitive functioning of ALS patients. We were particularly interested in verbal fluency and different aspects of executive functioning since these are commonly impaired in ALS patients and also have been connected to the cerebellum. We examined grey matter volume (GMV) and WM integrity of various supra- and infratentorial regions to find correlates of patients’ performance on different cognitive tasks as measured with a comprehensive neuropsychological battery.

## Materials and methods

### Ethics declarations

The study was carried out in accordance with the ethical standards laid down in the 1964 Declaration of Helsinki and its later amendments. Patients gave their written informed consent, and the study was approved by the local medical ethics committee at each site.

#### Participants

We analysed cross-sectional data from 120 patients recruited via the departments of neurology of the Rostock and Magdeburg University Medical Centres. Patients were classified according to the modified El Escorial criteria^26^ and a subsample of 11 cases additionally met the Rascovsky criteria for behavioural variant of frontotemporal dementia (bvFTD).^27^ The patients were characterized on the revised ALS functional rating scale (ALSFRS-R).^28^ The patients were categorized into familial and sporadic ALS based on family history. A group of 88 healthy controls (HCs) was recruited through public advertisements. All participants received a clinical and neurological examination and completed magnetic resonance imaging (MRI). Exclusion criteria included a history of brain injury, epilepsy or psychiatric illness.

#### Data acquisition

##### Neuropsychological assessment

Both patients as well as controls completed a comprehensive neuropsychological battery. Trail Making Test,^29^ Digit Span Forward and Backward,^30^ Tower of London^31^ and the computerized Stroop-Paradigm^32^ were used to assess executive functioning and attention. The scores used as outcome variables were not affected by the patients’ speech and motor impairments (use of ratios for the Trail Making Test and the Stroop-Paradigm, a choice of oral or written version of Digit Span Test, and a lack of time limitation for Tower of London). Phonetic and semantic verbal fluency were measured with the Regensburg Word Fluency Test,^33^ while accounting for patients’ speech and motor impairments by calculating a fluency index.^34^ The patients completed either the Verbal Learning and Memory Test^35^ or the short form of the California Verbal Learning Test^36^ as a memory assessment and the percentages of learned or recalled items were calculated to ensure the comparability of the different length tests. Not all patients managed to complete the whole neuropsychological battery due to severe motor impairments or fatigue.

Cognitive impairment was classified according to the revised Strong criteria,^37^ which define abnormal performance on a task as a score that is two or more standard deviations below the mean of HC. Taking into consideration missing data and multiple tests per cognitive domain, we applied the criteria for determining cognitive impairment as follows. The domain of executive functions was considered to be impaired when patients scored abnormal on at least two distinct executive tasks out of four. Verbal fluency was considered to be impaired when patients scored abnormal on at least one task out of four. Patients were divided into cognitively normal ALS patients (ALSni) and cognitively impaired ALS patients (ALSci). The patients that fulfilled the criteria for the behavioural variant of frontotemporal dementia (ALS-bvFTD) were considered as a separate group.

##### Magnetic resonance imaging

Data were acquired with identical settings and parameters in Rostock and Magdeburg on 3T Siemens MAGNETOM Verio scanners. Both sites fulfilled the common criteria in the phantom tests.^38,39^ High-resolution T1-weighted anatomical images were acquired using a magnetization-prepared rapid gradient echo (MPRAGE) sequence with the following parameters: repetition time = 2500 ms, echo time = 4.82 ms, flip angle 7, image matrix = 256 × 256, field of view = 250 × 250 × 192 mm, 192 sagittal slices and isotropic resolution = 1 mm^3^. The diffusion-weighted images were acquired using a twice refocused, spinecho echoplanar sequence: repetition time = 12 700 ms, echo time = 81 ms, parallel imaging with GRAPPA factor = 3, isotopic resolution = 2.0 mm^3^, field of view = 256 × 256, acquisition matrix = 128 × 128, 72 slices, 1 non-diffusion weighted and 30 non-collinear diffusion gradient directions, *b*-value = 1000 s/mm^2^, two averages and total scan time = 14:10 min.

#### MRI preprocessing

##### Grey matter volume

GM preprocessing was carried out using the cat12 toolbox^40^ and the Spatially Unbiased Infratentorial toolbox (SUIT)^41^ implemented in SPM12 (https://www.fil.ion.ucl.ac.uk/spm/). The T_1_-weighted MRIs were skull-stripped, segmented and the GM segment was spatially normalized to the MNI152 non-linear asymmetric 2009 template using Dartel registration in the cat12 toolbox. Modulation was applied to preserve volume and an 8 mm full-width at half maximum kernel was used for smoothing. The cerebellum GM was preprocessed with the SUIT toolbox since it features a high-resolution unbiased cerebellum atlas template and offers automated isolation and segmentation of the cerebellum. Each individual cerebellar mask was inspected for quality, and if necessary, the mask threshold was altered. Subsequently, the extracted cerebellar GM was normalized to the SUIT atlas template space and resliced with modulation to preserve volume. The AAL atlas^42^ was used to extract the GMV in supratentorial ROIs, whereas the SUIT parcellation atlas^43^ was used for the cerebellum. Cerebellar lobular volumes were calculated by summing the lobular GM in both hemispheres and vermis. The anterior and posterior cerebellum volumes were calculated by summing lobules I-V and VI-X, respectively. Lastly, the global cerebellar volume was calculated.

##### Diffusion-weighted images

The diffusion datasets were processed with the FSL software package (www.fmrib.ox.ac.uk/fsl). Preprocessing included correction for eddy currents and motion, and brain-tissue extraction. Then, a tensor model was fitted with FSL’s dtifit and fractional anisotropy (FA) was estimated. We settled on estimating FA as the only measure of WM integrity to avoid the problem of collinearity in the statistical analyses. The FA maps were processed using the Tract-Based Spatial Statistical tool.^44^ The individual FA maps were registered to the mean FA image from the Johns Hopkins University (JHU) WM atlas. The individual FA images were averaged to generate the mean FA image and a threshold of 0.3 was used to create the mean FA skeleton. The JHU ICBM-DTI-81 WM atlas^45^ was used to extract average FA values for supra- and infratentorial ROIs from skeletonized maps using custom Matlab code.

#### Statistical analysis

##### Neuropsychological and clinical data

The statistical analyses were completed in the Statistical Product and Service Solutions (SPSS, version 28.0). The whole sample was characterized by various demographic and clinical variables. The equivalence of the cognitive groups was compared using chi-squared tests for categorical variables and analysis of variance (ANOVA) for continuous variables. The groups were compared on neuropsychological data using analysis of covariance (ANCOVA) with years of education as a covariate and Bonferroni correction was used for the *post hoc* pairwise comparisons.

##### Imaging data

The statistical analyses were completed in SPSS and R (https://www.r-project.org/). The code used for the analyses can be found in the Supplementary Materials (elastic_net_bootstrap.R). Regression analyses were used to identify regions where GMV, mean FA values or information offered by demographical or clinical variables explained neuropsychological scores. The complete list of predictor variables can be found in Table 1. We chose a comprehensive list of GM regions and WM tracts that have been suggested to be related to cognitive functions in ALS samples, based on previous studies,^12,46^ spanning the frontal, temporal and parietal cortex in addition to subcortical areas. To pinpoint the specific contributions of cerebellar subregions, specialized regions needed to be considered. The use of many predictor variables brings challenges that have to be addressed, such as multicollinearity (e.g. correlations between the neighbouring regions metrics) and predictor selection (e.g. only considering the predictor variables that are informative for the outcome variable).

**Table 1:** The complete list of predictor variables entered into the elastic net regularized regression models

Grey matter volume	Mean fractional anisotropy	Demographic and clinical variables
Superior frontal gyrus	Corpus callosum genu	Education
Middle frontal gyrus	Corpus callosum body	Disease progression rate
Inferior frontal gyrus	Corpus callosum splenium	Sex
Medial superior frontal gyrus	Fornix	Age
Ventromedial prefrontal cortex	Anterior limb of internal capsule	
Orbitofrontal cortex	Anterior corona radiata	
Insula	Superior corona radiata	
Anterior cingulate cortex	Posterior thalamic radiation	
Hippocampus	External capsule	
Parahippocampal gyrus	Cingulum (cingulate gyrus)	
Supramarginal gyrus	Cingulum (hippocampus)	
Precuneus	Fornix cres/Stria terminalis	
Caudate nucleus	Superior longitudinal fasciculus	
Putamen	Uncinate fasciculus	
Pallidum	Superior cerebellar peduncle	
Superior temporal gyrus	Middle cerebellar peduncle	
Middle temporal gyrus	Inferior cerebellar peduncle	
Temporal pole		
Inferior temporal gyrus		
Cerebellar lobule V		
Cerebellar lobule VI		
Cerebellar crus I		
Cerebellar crus II		
Cerebellar lobule VIIb		
Cerebellar lobule VIIIa		
Cerebellar lobule VIIIb		
Cerebellar lobule IX		
Cerebellar lobule X		

To tackle both the expected correlations between predictor variables (addressed by the ridge penalty term) and the necessity of predictor selection (addressed by the lasso penalty term), elastic net model was chosen as a type of regularized regression.^47,48^ In more detail, the predictor variables were standardized and the regression coefficients were estimated through an automated tuning process (caret package in R). The best-fitting model featuring the smallest root mean squared error (RMSE) was determined through a 10-fold cross-validation and the model R square (*R*^2^) was extracted.

This model building process followed the bootstrap procedure for each cognitive outcome (1000 repeats with replacement).^49,50^ This method allowed us to estimate the variability in the regression coefficient estimates, model fit and the predictor variables retained in the final models. However, applying bootstrapping in regularized regression also comes with drawbacks that need to be considered when interpreting results. First, the bootstrapping procedure cannot estimate the bias that is inherently introduced into the regression coefficients through regularization. Regularization shrinks the regression coefficients towards zero, which leads to lower coefficient values and this effect remains despite bootstrapping. Second, tuning the penalty parameters on each bootstrap sample can yield widely varying models both in terms of the collection of retained predictor variables and their regression coefficient values. Nevertheless, bootstrapping can be used as a valuable tool in elastic net regression, particularly for understanding the variability of model fit and coefficient estimates in addition to assessing the stability of predictor variables selection.

For each cognitive outcome variable, we calculated the median *R*^2^ and its 90% confidence interval based on the 1000 models from the bootstrap samples. A high median *R*^2^ value for a cognitive outcome indicates that a large proportion of variation in the outcome variable can be explained by the models. Contrarily, a low median *R*^2^ value suggests that the available predictor variables do not explain the variation in the outcome variable very well. A wide-ranging confidence interval for *R*^2^ indicates that the explanatory power of the models differs greatly from bootstrap sample to sample, whereas a narrow confidence interval suggests that the model explains a similar amount of variation across samples.

To examine predictor variables selection, we estimated predictor stability and regression coefficient distributions. We calculated predictor stability as the proportion of bootstrap samples where the predictor variable was included in the final model (percentage of non-zero regression coefficients across all 1000 bootstrap samples). High stability indicates a consistent, reproducible effect across the available data. Furthermore, we determined the regression coefficient distributions by aggregating the non-zero coefficients for each parameter across the 1000 bootstrap samples and calculating the median and the 90% confidence interval. A wide regression coefficient distribution indicates that the meaningfulness of the predictor variable varies greatly with sample composition.

In addition, to further inquire about possible group differences in cerebellar structure, the global and lobular cerebellar GMV were compared between HC, ALSni, ALSci and ALS-bvFTD patients with ANCOVAs featuring age, sex and total intracranial volume (TIV) as covariates. Similarly, the mean FA of the cerebellar peduncles was compared between HC, ALSni, ALSci and ALS-bvFTD patients with ANCOVAs including age and sex as covariates.

## Results

### Clinical and neuropsychological assessment

The demographic and clinical details of our sample can be found in Table 2. The patient groups were compared on available cognitive data (Table 3). All groups differed in verbal fluency performance. ALS-bvFTD patients had lower scores on memory variables than ALSni and ALSci patients, and ALSni had higher scores on the executive task of digit span as compared with both ALSci and ALS-bvFTD patients.

**Table 2:** The equivalence of groups on demographic and clinical variables

	HC	ALSni	ALSci	ALS-bvFTD	Total	*P*-value^a^
Group size: count	88	68	41	11	208	
Age: M (SD)	60.6 (10.5)	59.8 (11.3)	58.6 (10.9)	62.8 (9.6)	60.0 (10.8)	0.626
Sex: Female/Male	33/55	21/47	19/22	4/7	77/131	0.451
Education years: M (SD)	13.7 (2.1)	13.6 (2.8)	12.2 (1.7)	12.5 (2.1)	13.3 (2.3)	**0**.**004**
Months since onset: median (IQR)		17 (10.0–32.0)	15 (10.0–36.0)	12 (8.5–15.0)	16 (9.8–32.0)	0.694
ALSFRS-R: median (IQR)		41.0 (34.0–42.0)	38.0 (36.0–43.0)	43.0 (40.0–45.0)	40.0 (35.0–43.0)	0.241
Progression rate^b^: median (IQR)		0.5 (0.3–0.9)	0.4 (0.2–0.7)	0.4 (0.2–0.6)	0.4 (0.2–0.8)	0.662
Site of onset: count (%)						0.089
Spinal onset		31 (46%)	22 (54%)	3 (27%)	56 (47%)	
Bulbar onset		25(37%)	16 (39%)	8 (73%)	49 (41%)	
Unknown		12 (17%)	3 (7%)	0	15 (12%)	
Phenotype: count						0.576
Classical ALS		48	25	7	80	
PLS		5	3	0	8	
UMND		4	4	3	11	
PMA		3	3	1	7	
LMND		8	5	0	13	
Not classifiable		0	1	0	1	
Genetic status: count						0.526
Familial		4	1	0	5	
Sporadic		64	39	11	114	
Unknown		0	1	0	1	

*P*-values < 0.05 in bold.

ALSci, cognitively impaired; ALSni, not cognitively impaired; ALSFRS-R, revised ALS functional rating scale; ALS-bvFTD, concurrent behavioural variant of frontotemporal dementia; HC, healthy controls; IQR, interquartile range; LMND, lower motor neuron dominant ALS; M, mean; PLS, primary lateral sclerosis; PMA, progressive muscular atrophy; SD, standard deviation; UMND, upper motor neuron dominant ALS.

^a^Testing for group differences with Chi-squared tests for categorical and ANOVA for quantitative variables.

^b^Monthly decrease rate in the ALSFRS-R score.

**Table 3:** Comparison of patient groups on neuropsychological scores

Dependent variable	ALSni		ALSci		ALS-bvFTD		*P*-value^a^		
	*N*	M^b^ (SD)	*N*	M^b^ (SD)	*N*	M^b^ (SD)	ALSni versus ALSci	ALSni versus ALS-bvFTD	ALSci versus ALS-bvFTD
Executive functions									
Digit span	67	−0.35 (0.9)	41	−0.99 (0.9)	10	−1.63 (0.7)	**0**.**020**	**0**.**001**	0.053
Trail Making Test	63	−0.03 (0.9)	35	0.35 (1.4)	7	0.98 (1.2)	0.663	0.078	0.388
Stroop Test	51	−0.27 (1.4)	31	−0.44 (1.4)	7	−0.29 (1.6)	1.000	1.000	1.000
Tower of London	36	−0.56 (0.8)	21	−0.27 (1.0)	4	0.27 (1.4)	1.000	0.330	0.746
Verbal fluency									
Letter fluency	52	0.74 (1.8)	38	2.82 (2.7)	7	6.38 (3.2)	**0**.**005**	**0**.**001**	**0**.**001**
Semantic fluency	52	−0.33 (0.8)	38	0.58 (1.2)	6	2.95 (1.4)	**0**.**005**	**0**.**001**	**0**.**001**
Memory									
Learning sum	65	−0.44 (1.1)	41	−0.68 (1.2)	10	−2.35 (1.0)	1.000	**0**.**001**	**0**.**001**
Immediate recall	65	−0.15 (1.2)	41	−0.33 (1.3)	9	−1.09 (2.1)	1.000	0.186	0.365
Delayed recall	65	−0.28 (0.9)	41	(−0.55 (1.2)	8	−1.93 (1.7)	1.000	**0**.**001**	**0**.**001**

*P*-values < 0.05 in bold.

ALSci, cognitively impaired; ALSni, not cognitively impaired; ALS-bvFTD, concurrent behavioural variant of frontotemporal dementia; M, mean; SD, standard deviation.

^a^Bonferroni-corrected.

^b^Mean expressed as the *z*-score calculated based on scores from healthy controls.

### Structural imaging

**Figure 1:**
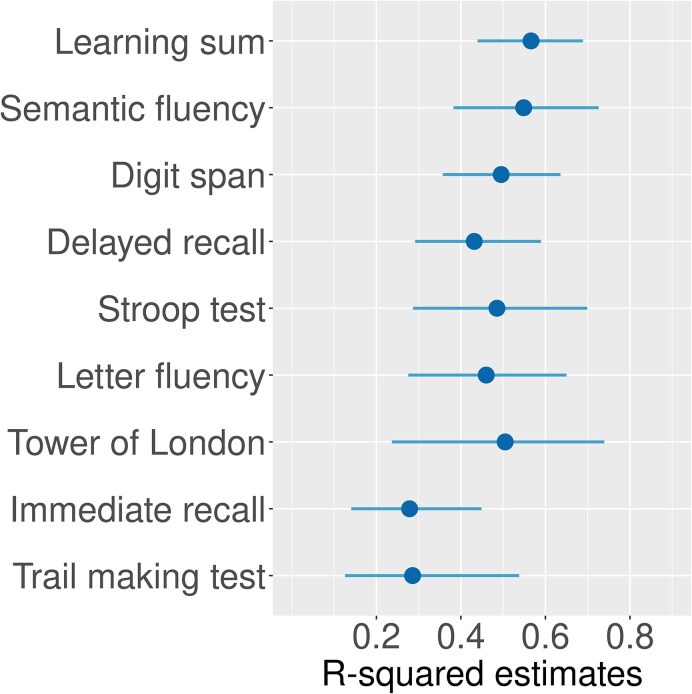
**The median and bootstrapped 90% confidence interval of model *R*^2^ values per outcome variable.** For each cognitive outcome variable, the *R*^2^ value of the best-fitting model for each bootstrap sample (*N* = 1000) was calculated. The distributions show the median and the 90% confidence interval of the compiled *R*^2^ values.

The variation in the *R*^2^ values, which describes how well the models per cognitive outcome fit the data, is illustrated in Fig. 1 and the numerical values can be found in Supplementary Table 1. The wide confidence intervals for some cognitive outcome variables, e.g. *R*^2^ ranging from 0.24 to 0.74 for Tower of London, indicate that model fit differed greatly. In other words, the informativeness of the models for the same outcome variable fluctuated a lot from one sample to the other. Other cognitive outcome variables could be modelled more stably and their confidence intervals for *R*^2^ values were narrower, e.g. *R*^2^ ranging from 0.44 to 0.69 for learning sum. Nevertheless, the relatively high median *R*^2^ values, e.g. 0.57 for learning sum, 0.55 for semantic fluency, and 0.50 for digit span, indicate that the regularized regression analyses detected informative predictor variables.

**Figure 2:**
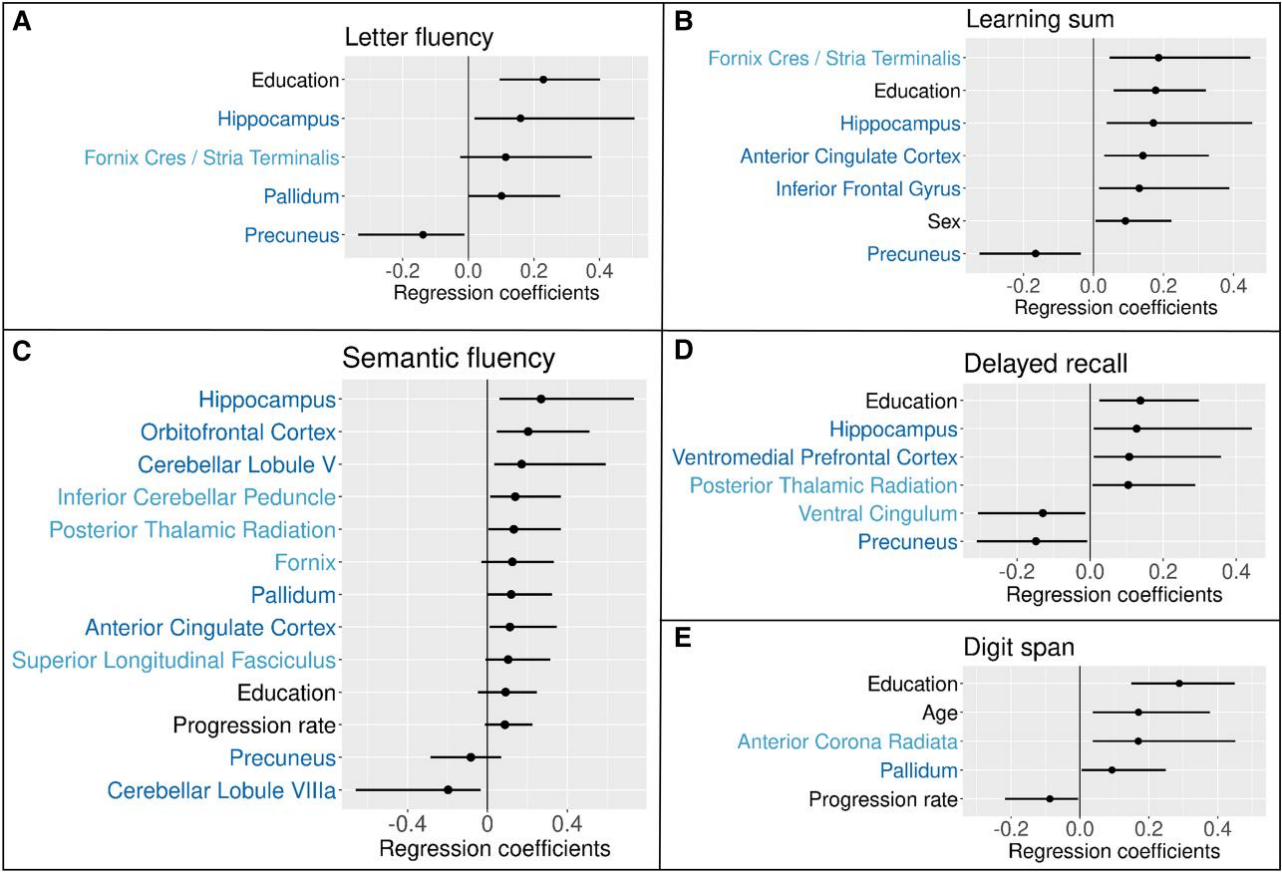
**The most reliable predictor variables of cognitive scores based on a bootstrapping procedure.** The regression coefficients of predictor variables from elastic net regularized regression analyses for letter fluency (panel **A**, *N* = 96), learning sum (panel **B**, *N* = 120), semantic fluency (panel **C**, *N* = 93), delayed recall (panel **D**, *N* = 118) and digit span (panel **E**, *N* = 120). All predictor variables were included in over 80% of the models fitted to the 1000 bootstrap samples per cognitive outcome. The distribution of regression coefficient values across the models is illustrated by the median and the 90% confidence interval. The demographic variables are in black, regional GMV in dark blue and regional FA in light blue.

For selected cognitive outcomes, the predictor variables that were included in over 80% of the models are listed with their median regression coefficient value and the 5th and 95th percentile in Table 4. The predictor variables for all cognitive outcomes are summarized in Supplementary Table 2. The most stable predictor variables for selected models are visualized in Fig. 2. Years of education had a strong association with cognitive scores in general since it predicted cognitive performance in 8 of the 9 outcomes with over 80% stability. Pallidum volume was positively correlated with fluency outcomes and working memory. Hippocampus volume was positively related to fluency and memory outcomes, but the WM FA of the ventral cingulum leading to the hippocampus was negatively related to the memory outcomes. Interestingly, precuneus volume was negatively associated with fluency and memory outcomes. The GMV of cerebellar lobules V and VIIIa featured consistently as explanatory variables of semantic fluency. No other associations emerged between cerebellar regions and cognitive outcomes.

**Table 4:** The predictor variables that were included in over 80% of the best-fitting models based on bootstrap samples (*N* = 1000) with their median and the 5th and 95th percentile regression coefficient values

Outcome variables	Predictor variables	Stability (%)	Median	5%	95%
Digit span	Education	100	0.289	0.149	0.450
	Anterior corona radiata	94.9	0.169	0.037	0.451
	Progression rate	88.1	−0.087	−0.218	−0.005
	Age	87.9	0.170	0.037	0.377
	Pallidum	81.1	0.093	0.005	0.250
Letter fluency	Education	99.5	0.229	0.096	0.401
	Precuneus	88.8	−0.138	−0.335	−0.012
	Pallidum	86.3	0.101	0.001	0.280
	Hippocampus	84.2	0.159	0.018	0.507
	Fornix cres/stria terminalis	80.5	0.114	−0.025	0.376
Semantic fluency	Hippocampus	97.1	0.269	0.060	0.734
	Orbitofrontal cortex	96.3	0.204	0.047	0.512
	Cerebellar lobule V	95.3	0.172	0.034	0.593
	Cerebellar lobule VIIIa	91.7	−0.197	−0.660	−0.035
	Progression rate	90.1	0.088	−0.013	0.226
	Inferior cerebellar peduncle	89.2	0.139	0.014	0.368
	Pallidum	89	0.119	0.001	0.324
	Fornix	87.2	0.125	−0.030	0.333
	Education	86.4	0.091	−0.047	0.248
	Anterior cingulate cortex	85.8	0.113	0.011	0.347
	Superior longitudinal fasciculus	85.7	0.104	−0.010	0.315
	Posterior thalamic radiation	83.1	0.132	0.005	0.369
	Precuneus	80.7	−0.083	−0.286	0.070
Learning sum	Education	99.1	0.177	0.057	0.322
	Fornix cres/stria terminalis	98.3	0.186	0.045	0.448
	Hippocampus	96	0.171	0.038	0.454
	Anterior cingulate cortex	95.3	0.141	0.030	0.329
	Precuneus	91.8	−0.165	−0.325	−0.036
	Inferior frontal gyrus	88.6	0.131	0.015	0.388
	Sex	86.2	0.091	0.006	0.223
Delayed recall	Education	93	0.138	0.025	0.297
	Cingulum (hippocampus)	86.4	−0.129	−0.307	−0.013
	Precuneus	85.5	−0.148	−0.310	−0.008
	Hippocampus	84.1	0.127	0.010	0.442
	Ventromedial prefrontal cortex	82.5	0.107	0.010	0.358
	Posterior thalamic radiation	81.9	0.104	0.006	0.288

In group comparison analyses with global and lobular cerebellar GMV, no significant differences were observed between HC, ALSni, ALSci and ALS-bvFTD (Supplementary Table 3). The ALS-bvFTD patients had lower FA values in both superior and inferior cerebellar peduncles compared with HC, ALSni and ALSci (Bonferroni-corrected *P*-values of 0.004, 0.001, 0.005 and 0.001, 0.001 and 0.012, respectively). The other comparisons were not significant (Supplementary Table 3).

## Discussion

In this study, we considered both GMV and WM integrity in cerebellar and cerebral regions to investigate their comparative influence on the cognitive functioning of ALS patients. We used elastic net regression and bootstrapping to evaluate the reliability of regional metrics and to capture the variability in the estimated regression coefficients per cognitive outcome. Our primary finding was the association of specific cerebellar lobule volumes with semantic fluency, whereas all other cerebellar GM or WM metrics were not reliably related to any of the other cognitive measures. Most of the cognitive scores examined in this study were associated with a combination of cortical and subcortical GMVs in addition to the integrity of various WM tracts.

The involvement of cerebellar structures in verbal fluency is very plausible. Previous research has suggested that cerebellum plays a role in acquiring novel strategies for processing sequenced information such as organizing a lexical search.^51^ Contrary to our expectations, however, cerebellum regional metrics were only related to semantic fluency scores and not to phonemic fluency scores. Furthermore, the limited importance of cerebellum for other cognitive functions suggested by our findings is compatible with the heterogeneous results from previous studies investigating associations between cerebellar involvement and cognition in ALS. Some studies reported no differences in cerebellar regional volumes^19,52^ or WM integrity across different cognitive profiles.^53^ One investigation detected increased cerebellar brain volume in cognitively normal ALS patients compared with HCs, which was interpreted by the authors as a potential resilience factor.^54^ Other publications have described a GMV reduction in the left cerebellar lobule VIII in ALS patients with cognitive or behaviour changes,^17^ found general cognitive functioning to be correlated with the cerebellar superior lobe and crus GMV,^55^ and verbal fluency to be related to the cerebellar lobule VI and crus GMV.^19^ These incongruences in the literature may stem from the application of diverse neuropsychological assessments (e.g. differently composed test batteries^17,19,53^ or screening tools like Addenbrook’s Cognitive Estimation).^52,55^ Furthermore, inconsistencies may arise from applying different study designs or statistical approaches (e.g. comparison of group profiles from principal component analysis,^19^ correlational analyses,^52^ univariate ANOVA,^53^ whole-brain voxel-based analysis^17^ or voxel-based analysis restricted to the cerebellum^55^) to inherently heterogeneous samples. Nevertheless, the general pattern of results does not indicate strong associations between the regional GMV and WM integrity and the performance of ALS patients in the majority of cognitive domains.

As expected, our analyses also highlighted various non-cerebellar regions where the GM or WM metrics were related to cognitive scores. For instance, the GMV of the pallidum showed a positive correlation with both fluency and working memory, and hippocampal volume was positively associated with fluency and various memory outcomes. Unsurprisingly, educational achievement emerged as the most general and reliable predictor of cognitive performance on the neuropsychological tests^56^ and thus calls for a detailed consideration of further variables beyond structural metrics that contribute to the individual variability in cognitive performance.

The current methodological approach combining regularized regression and bootstrapping allowed us to investigate the contributions of numerous correlated predictors and to quantify their reliability across differently composed samples. However, despite the theoretic suitability of elastic net regression for these analyses, the statistical approach still seemed to reach its limits due to associations between predictors. In our results, the GMV of precuneus was negatively associated with memory and fluency outcomes in contrast to the wider literature^57^ and the FA of ventral cingulum connecting to the hippocampus was also negatively related to delayed memory recall, which is inconsistent with previously published data.^58^ For semantic fluency, cerebellar lobule V had a positive regression coefficient, whereas cerebellar lobule VIIIa had a negative one. These incongruent findings highlight the potential shortcomings of statistical models in situations with complex covariance structures between the predictors.^59,60^

Our neuropsychological test battery, adapted to accommodate the motor impairments of ALS patients, offered a detailed assessment of cognitive functions and enabled a classification of patients according to the revised Strong criteria^37^ and subsequent group comparisons. In accordance with this classification, the ALSci performed worse than ALSni on working memory, letter and semantic fluency tasks but not on episodic memory tasks. ALS-FTD patients, however, performed worse than ALSni in all domains (executive functioning, fluency and memory). Thus, the cognitive status in our sample is consistent with the literature.^12^

A few limitations need to be considered when interpreting the findings of the current study. First, to limit the problem of multicollinearity, the regional structural metrics included as predictors in the analyses were the average values of the bilateral regions. This approach, therefore, considered the regions of both hemispheres to be equivalent, which is a simplification. Second, regularization inherently introduces bias into the parameter estimates by shrinking the coefficients of correlated predictors towards zero. Therefore, in the current analyses, the coefficient values are estimated closer to zero than in non-regularized regression and do not lend themselves to a straightforward interpretation of effect size. This bias also cannot be captured by bootstrapping, which only reflects the variance in the biased estimates. Third, a larger sample would allow the consideration and juxtaposition of cognitive correlates in ALS subtypes for instance dependent on different neurodegenerative patterns.

In conclusion, cerebellar lobule volumes were associated with only semantic fluency performance, and cerebellar GM or WM metrics were not related to any of the other cognitive measures. Cognitive functioning in ALS patients was associated with a combination of cortical and subcortical GMVs in addition to the integrity of various WM tracts. Further investigations could focus on the involvement of the cerebellum in the affective and social-emotional dysfunction present in a proportion of ALS patients.

## Supplementary Material

fcaf230_Supplementary_Data

## Data Availability

The data analysed in the current study can be received from the corresponding author upon reasonable request from qualified investigators.
