# Supplementary material for: Cerebellar grey matter volume is associated with semantic fluency performance in amyotrophic lateral sclerosis patients
Source: Brain Commun. 2025 Jun 12;7(3):fcaf230. doi: 10.1093/braincomms/fcaf230 (PMC12199757; doi:10.1093/braincomms/fcaf230)
Supplement: fcaf230_Supplementary_Data [file fcaf230_supplementary_data.zip › Supplementary_tables.docx]

**Supplementary Material**

**Cerebellar grey matter volume is associated with semantic fluency performance in amyotrophic lateral sclerosis patients**

Annaliis Lehto^a,b*^, Julia Schumacher^b,c^, Stefan Teipel^b,d^, Judith Machts^e^, Stefan Vielhaber^e^, Andreas Hermann^a,b^, Johannes Prudlo^b,c^, Elisabeth Kasper^b, c*^

^a^ Translational Neurodegeneration Section “Albrecht Kossel”, Department of Neurology, Rostock University Medical Center, Rostock, Germany,

^b^ Deutsches Zentrum für Neurodegenerative Erkrankungen (DZNE), Rostock-Greifswald, Rostock, Germany,

^c^ Department of Neurology, Rostock University Medical Center, Rostock, Germany,

^d^ Department of Psychosomatic Medicine, Rostock University Medical Center, Rostock, Germany,

^e^ Otto von Guericke University Magdeburg, Department of Neurology, Magdeburg, Germany

|  |  | **R^2^** | | |
| --- | --- | --- | --- | --- |
| **Dependent variable** | **Sample size** | **Median** | **5 %** | **95 %** |
| Digit span | 120 | 0.495 | 0.357 | 0.636 |
| Trail making test | 100 | 0.286 | 0.126 | 0.538 |
| Stroop test | 83 | 0.485 | 0.287 | 0.699 |
| Tower of London | 61 | 0.505 | 0.236 | 0.739 |
| Letter fluency | 96 | 0.460 | 0.275 | 0.650 |
| Semantic fluency | 93 | 0.549 | 0.382 | 0.726 |
| Learning sum | 120 | 0.566 | 0.439 | 0.689 |
| Immediate recall | 119 | 0.279 | 0.141 | 0.449 |
| Delayed recall | 118 | 0.432 | 0.292 | 0.590 |

**Supplementary Table 1** The median and the 90% confidence interval of model R^2^ values for each cognitive outcome variable from 1000 bootstrapping samples

| **Outcome Variables** | **Predictor variables** | **Stability (%)** | **Median** | **5%** | **95%** |
| --- | --- | --- | --- | --- | --- |
| Digit Span | Education | 100 | 0.289 | 0.149 | 0.450 |
|  | Anterior Corona Radiata | 94.9 | 0.169 | 0.037 | 0.451 |
|  | Progression rate | 88.1 | -0.087 | -0.218 | -0.005 |
|  | Age | 87.9 | 0.170 | 0.037 | 0.377 |
|  | Pallidum | 81.1 | 0.093 | 0.005 | 0.250 |
| Trail Making Test | Cingulum (Hippocampus) | 93 | -0.192 | -0.521 | -0.060 |
| Stroop Test | Age | 96.3 | -0.248 | -0.558 | -0.036 |
|  | Education | 91.7 | 0.139 | 0.005 | 0.347 |
|  | Middle Frontal Gyrus | 90 | -0.347 | -0.791 | -0.047 |
|  | Corpus Callosum Body | 89.4 | -0.251 | -0.694 | -0.050 |
|  | Progression rate | 82.5 | -0.104 | -0.352 | 0.101 |
|  | Corpus Callosum Genu | 82.4 | 0.200 | 0.023 | 0.648 |
| Tower of London | Age | 96.3 | -0.248 | -0.558 | -0.036 |
|  | Education | 91.7 | 0.139 | 0.005 | 0.347 |
|  | Middle Frontal Gyrus | 90 | -0.347 | -0.791 | -0.047 |
|  | Corpus Callosum Body | 89.4 | -0.251 | -0.694 | -0.050 |
|  | Progression rate | 82.5 | -0.104 | -0.352 | 0.101 |
|  | Corpus Callosum Genu | 82.4 | 0.200 | 0.023 | 0.648 |
| Letter Fluency | Education | 99.5 | 0.229 | 0.096 | 0.401 |
|  | Precuneus | 88.8 | -0.138 | -0.335 | -0.012 |
|  | Pallidum | 86.3 | 0.101 | 0.001 | 0.280 |
|  | Hippocampus | 84.2 | 0.159 | 0.018 | 0.507 |
|  | Fornix Cres / Stria Terminalis | 80.5 | 0.114 | -0.025 | 0.376 |
| Semantic Fluency | Hippocampus | 97.1 | 0.269 | 0.060 | 0.734 |
|  | Orbitofrontal Cortex | 96.3 | 0.204 | 0.047 | 0.512 |
|  | Cerebellar Lobule V | 95.3 | 0.172 | 0.034 | 0.593 |
|  | Cerebellar Lobule VIIIa | 91.7 | -0.197 | -0.660 | -0.035 |
|  | Progression rate | 90.1 | 0.088 | -0.013 | 0.226 |
|  | Inferior Cerebellar Peduncle | 89.2 | 0.139 | 0.014 | 0.368 |
|  | Pallidum | 89 | 0.119 | 0.001 | 0.324 |
|  | Fornix | 87.2 | 0.125 | -0.030 | 0.333 |
|  | Education | 86.4 | 0.091 | -0.047 | 0.248 |
|  | Anterior Cingulate Cortex | 85.8 | 0.113 | 0.011 | 0.347 |
|  | Superior Longitudinal Fasciculus | 85.7 | 0.104 | -0.010 | 0.315 |
|  | Posterior Thalamic Radiation | 83.1 | 0.132 | 0.005 | 0.369 |
|  | Precuneus | 80.7 | -0.083 | -0.286 | 0.070 |
| Learning Sum | Education | 99.1 | 0.177 | 0.057 | 0.322 |
|  | Fornix Cres / Stria Terminalis | 98.3 | 0.186 | 0.045 | 0.448 |
|  | Hippocampus | 96 | 0.171 | 0.038 | 0.454 |
|  | Anterior Cingulate Cortex | 95.3 | 0.141 | 0.030 | 0.329 |
|  | Precuneus | 91.8 | -0.165 | -0.325 | -0.036 |
|  | Inferior Frontal Gyrus | 88.6 | 0.131 | 0.015 | 0.388 |
|  | Sex | 86.2 | 0.091 | 0.006 | 0.223 |
| Immediate Recall | Ventromedial Prefrontal Cortex | 90.8 | 0.147 | 0.020 | 0.431 |
|  | Corpus Callosum Genu | 88.4 | 0.158 | 0.019 | 0.547 |
|  | Cingulum (Hippocampus) | 82.6 | -0.142 | -0.349 | -0.015 |
| Delayed Recall | Education | 93 | 0.138 | 0.025 | 0.297 |
|  | Cingulum (Hippocampus) | 86.4 | -0.129 | -0.307 | -0.013 |
|  | Precuneus | 85.5 | -0.148 | -0.310 | -0.008 |
|  | Hippocampus | 84.1 | 0.127 | 0.010 | 0.442 |
|  | Ventromedial Prefrontal Cortex | 82.5 | 0.107 | 0.010 | 0.358 |
|  | Posterior Thalamic Radiation | 81.9 | 0.104 | 0.006 | 0.288 |

**Supplementary Table 2** The predictor variables that were included in over 80% of the best-fitting models based on bootstrap samples (N=1000) with their median and the 5^th^ and 95^th^ percentile regression coefficient values

|  |  |  | **HC – ALS-bvFTD** | | **ALSni – ALS-bvFTD** | | **ALSci – ALS-bvFTD** | |
| --- | --- | --- | --- | --- | --- | --- | --- | --- |
|  | **F-statistic** | **Sig.** | **Mean difference** | **Sig.** | **Mean difference** | **Sig.** | **Mean difference** | **Sig.** |
| **White matter FA** |  |  |  |  |  |  |  |  |
| MCP | 2.18 | 0.091 |  |  |  |  |  |  |
| ICP | 6.56 | <0.001 | 0.038 | <0.001 | 0.042 | <0.001 | 0.032 | 0.012 |
| SCP | 5.13 | 0.002 | 0.038 | 0.004 | 0.044 | <0.001 | 0.040 | 0.005 |
| **Grey matter volume** |  |  |  |  |  |  |  |  |
| VI | 0.88 | 0.451 |  |  |  |  |  |  |
| Crus I | 0.60 | 0.615 |  |  |  |  |  |  |
| Crus II | 0.89 | 0.448 |  |  |  |  |  |  |
| VIIb | 0.52 | 0.669 |  |  |  |  |  |  |
| VIIa | 0.73 | 0.535 |  |  |  |  |  |  |
| VIIIb | 1.44 | 0.234 |  |  |  |  |  |  |
| IX | 1.71 | 0.165 |  |  |  |  |  |  |
| X | 1.53 | 0.208 |  |  |  |  |  |  |

**Supplementary Table 3** The group differences for cerebellar peduncles and lobules from univariate analyses of covariance with covariates age and sex

ALSci, cognitively impaired; ALSni, not cognitively impaired; ALS-bvFTD, concurrent behavioural variant of frontotemporal dementia; FA, fractional anisotropy; HC, healthy controls; ICP, inferior cerebellar peduncle; MCP, middle cerebellar peduncle; SCP, superior cerebellar peduncle; sig, significance.
